# Physiotherapists’ barriers and facilitators to the implementation of a behaviour change-informed exercise intervention to promote the adoption of regular exercise practice in patients at risk of recurrence of low back pain: a qualitative study

**DOI:** 10.1186/s12875-024-02274-y

**Published:** 2024-01-26

**Authors:** Alexandre Moniz, Susana T. Duarte, Pedro Aguiar, Carmen Caeiro, Diogo Pires, Rita Fernandes, Diogo Moço, Marta M. Marques, Rute Sousa, Helena Canhão, Jaime Branco, Ana Maria Rodrigues, Eduardo B. Cruz

**Affiliations:** 1 Comprehensive Health Research Center (CHRC), NOVA Medical School|Faculdade de Ciências Médicas, NMS|FCM, Universidade NOVA de Lisboa, Lisbon, Portugal; 2 EpiDoc Unit, NOVA Medical School|Faculdade de Ciências Médicas, NMS|FCM, Universidade Nova de Lisboa, Lisbon, Portugal; 3 Departamento de Fisioterapia, Escola Superior de Saúde, Instituto Politécnico de Setúbal, Setúbal, Portugal; 4 Comprehensive Health Research Center (CHRC), National School of Public Health, Universidade NOVA de Lisboa, Lisbon, Portugal; 5 National School of Public Health, Universidade NOVA de Lisboa, Lisbon, Portugal; 6 Comprehensive Health Research Center (CHRC), Universidade NOVA de Lisboa, Lisbon, Portugal; 7 Serviço de Reumatologia Do Hospital Egas Moniz, Centro Hospitalar Lisboa Ocidental (CHLO), Lisbon, Portugal; 8 Rheumatology Unit, Hospital Dos Lusíadas, Lisbon, Portugal

**Keywords:** Low back pain, Exercise, Behaviour change, Implementation science, Primary healthcare, Qualitative research

## Abstract

**Background:**

Recurrences of low back pain (LBP) are frequent and associated with high levels of disability and medical costs. Regular exercise practice may be an effective strategy to prevent recurrences of LBP, however, the promotion of this behaviour by physiotherapists seems to be challenging. This study aims to explore physiotherapists’ perceived barriers and facilitators to the implementation of a behaviour change-informed exercise intervention to promote the adoption of regular exercise practice by patients at risk of recurrence of low back pain.

**Methods:**

Two focus groups with primary healthcare physiotherapists were conducted, based on a semi-structured interview schedule informed by the Behaviour Change Wheel, including the Capability, Opportunity, Motivation–Behaviour (COM-B) model and the Theoretical Domains Framework (TDF). All focus groups were held through videoconference, audio and video recorded and transcribed verbatim. A deductive content analysis, using a coding matrix based on the COM-B and TDF, was performed by two independent researchers. A third researcher was approached to settle disagreements.

**Results:**

In total, 14 physiotherapists participated in the focus groups. The analysis revealed a total of 13 barriers (4 COM-B components and 7 TDF domains) and 23 facilitators (5 COM-B and 13 TDF) to physiotherapists’ implementation of a behaviour change-informed exercise intervention. The most common barriers were the lack of skills and confidence to implement the proposed intervention. These were explained by the fact that it differs from the usual practice of most participants and requires the learning of new skills applied to their contexts. However, for those who had already implemented other similar interventions or whose rationale is aligned with the new intervention, there seemed to exist more positive determinants, such as potential benefits for physiotherapists and the profession, improvement of quality of care and willingness to change clinical practice. For others who did not previously succeed in implementing these types of interventions, more context-related barriers were mentioned, such as lack of time to implement the intervention, schedule incompatibilities and lack of material and human resources.

**Conclusions:**

This study identified modifiable barriers and facilitators to physiotherapists’ implementation of a behaviour change-informed exercise intervention for patients at risk of recurrence of LBP in primary healthcare. The findings of this study will allow the systematic and theory-based development of a behaviour change-informed training programme, aimed at physiotherapists and supporting the successful implementation of the exercise intervention.

**Supplementary Information:**

The online version contains supplementary material available at 10.1186/s12875-024-02274-y.

## Background

Low back pain (LBP) is the leading cause of years lived with disability (YLDs) worldwide, being responsible for approximately 568.4 million prevalent cases and 63.7 million YLDs [1]. This represents an increase of 47% since 1990 [1, 2].

The course of an LBP episode is typically favourable, with most people experiencing significant improvements in pain and disability after six weeks, and full recovery at 12 weeks [3]. However, recurrences of LBP are frequent, and some people even develop persistent and disabling pain [4]. A systematic review, investigating the risk of recurrence in people who recovered from an LBP episode, reported a 1-year recurrence proportion of 33% [5]. Another study found that within 12 months of recovery after an LBP episode, 69% of participants had a recurrence of LBP. Of those, 40% had a recurrence that limited activity and 41% resorted to healthcare [6]. These recurrences of LBP may be associated with greater disability and medical costs [7], being one of the major contributors to the burden of LBP worldwide [4].

While most effectiveness studies focus on the immediate management of LBP [8–13], evidence about effective strategies to prevent recurrences of LBP is scarce [14, 15]. A systematic review reported moderate-quality evidence that post-treatment exercise programmes can prevent recurrences of LBP [16]. Another systematic review with meta-analysis, investigating the effectiveness of interventions for the prevention of future LBP episodes, found that in the short-term (< 1 year), exercise alone could reduce 35% the risk of an LBP episode and 78% of the risk for sick leave, while exercise in combination with education presented a 45% risk reduction for a new recurrence [17]. This study also indicates that in the long-term (> 1 year), the effect size of exercise and education decreased, while it disappeared for exercise alone, suggesting that long-term adoption to exercise may be important for it to continue providing a protective effect [17]. These findings corroborate the adoption of regular exercise as an important self-management strategy and suggest that it might be an important behavioural target to prevent future recurrences of LBP, but difficult to achieve since about 50–70% of people with LBP do not adopt exercise in the long-term [18].

Behaviours that are undertaken by individuals to self-manage their health conditions and improve their own health outcomes are importantly influenced by healthcare professionals, namely physiotherapists (PTs) [19]. Like in other health professions, changing practices or integrating high-value care evidence practices seems to be challenging for PTs [20]. Recent evidence from systematic reviews indicates that the majority of PTs do not follow the recommendations of the most recent evidence-based guidelines in the management of LBP patients [20], or use a small number of behaviour change techniques to promote physical activity [21]. These findings suggest that to facilitate the desired behaviour change at the patient level and promote the regular adoption of exercise, a change in PTs’ behaviours and practice might also be required, and the implementation success of high-value care evidence practices will depend on it. This raises the need not only to develop effective and evidence-based interventions at the patient level aimed at the prevention of recurrences of LBP but also, to ensure that PTs receive proper training and increase their competency in effectively delivering the intervention as intended [22, 23].

Previous studies also indicate that change is more likely to be successful if interventions and implementation strategies are specifically designed to address pre-identified behavioural determinants [24, 25]. Therefore, there has been a growing interest in the use of theories, models and frameworks to inform implementation and understand what factors may determine its success [26]. Understanding the determinants for change and developing strategies that target them are key implementation principles, which can be facilitated by the use of theory [27]. These determinants, defined as “factors that obstruct (i.e., barriers) or enable (i.e., facilitators) changes in targeted professional behaviours or healthcare delivery processes” [28], can directly impact the implementation of new practices and influence health professionals’ desired behaviour change towards those practices [29].

Previous qualitative findings suggest the existence of several factors influencing healthcare professionals’ implementation of interventions focused on the promotion of physical activity-related behaviours, such as health professionals’ knowledge, attitudes, beliefs about capabilities, and training to deliver the interventions, among others [30]. Given the existence of multiple factors that may influence the quality of care, a comprehensive understanding of the specific setting and of what may hamper or enable PTs’ implementation of high-value care evidence practices is needed [31].

Therefore, this study aims to explore, from the PTs’ perspectives, the potential barriers and facilitators to the implementation of a behaviour change-informed exercise intervention to promote the adoption of regular exercise practice in patients at risk of recurrence of LBP. This information will subsequently guide the development of a training programme, to support the delivery of the behaviour change-informed exercise intervention by PTs.

## Methods

### Study design

A qualitative study, through focus groups (FGs) with PTs working in primary healthcare, was conducted to explore and identify barriers and facilitators to PTs’ implementation of a behaviour change-informed exercise intervention for patients at risk of recurrence of LBP. The Consolidated Criteria for Reporting Qualitative Research (COREQ) (Additional file 1) was used to guide the study design and the subsequent data analysis [32]. Ethical approval was granted by the Specialised Ethics Committee for Research from the School of Health, Polytechnic Institute of Setúbal (CEEI-ESS) (Reference 77/AFP/2021).

### Theoretical frameworks

The Behaviour Change Wheel (BCW), including the Capability (C) Opportunity (O) Motivation (M) – Behaviour (B) (COM-B) model and the Theoretical Domains Framework (TDF), was used to guide the interview schedule and the analysis of the barriers and facilitators. The BCW [33] allows for a comprehensive development process, through the identification of relevant determinants of behaviour and possible mechanisms of change, the development of effective strategies to design successful interventions, and helps to understand the success or failure of interventions and implementation strategies [34, 35]. At the core of the BCW sits the COM-B model, which endorses that behaviour is influenced by several components (capability, motivation and opportunity), which are essential for it to occur [36]. Additionally, the TDF can also be used as a way of reinforcing the analysis made by the COM-B model and provides a deeper understanding of the factors influencing the behaviours and how to target them within an intervention [37]. The TDF consists of 14 domains (*Knowledge*; *Skills*; *Social/professional role and identity*; *Beliefs about capabilities*; *Optimism*; *Beliefs about consequences*, *Reinforcement*; *Intentions*; *Goals*; *Memory, attention and decision processes*; *Environmental context and resources*; *Social influences*; *Emotions*; *Behavioural regulation*) [38], which can be directly linked to the COM-B components [33].

### Setting

The Portuguese National Health Service is a universal tax-financed system, with the Ministry of Health being responsible for the coordination of health care provision and financing [39]. At a regional level, the National Health Service is supervised by regional health administrations (RHAs), responsible for the management of groups of primary healthcare centres (ACES) [39]. In Portugal, public primary healthcare is mostly delivered through the ACES, which are composed of several units and have the mission of guaranteeing primary care services to the population of a specific geographical area [39].

Four of these ACES, which belong to two RHAs, participated in this study. Three (Arrábida, Arco Ribeirinho and Almada Seixal), belong to the Regional Health Administration of Lisbon and Tagus Valley (RHALTV). The other ACES (Alentejo Central) belongs to the Regional Health Administration of Alentejo (RHAA).

The ACES Arrábida provides health services to a population of 243.683 individuals, within three municipalities. The ACES Arco Ribeirinho, provides health services to a population of 210.884 individuals, living in four municipalities, while the ACES Almada Seixal, to a population of 366.165 individuals, from two municipalities. Finally, the ACES Alentejo Central provides health services to twelve municipalities with a total population of 167.980 individuals [40].

### Participants

PTs from the ACES previously described were purposively selected for participation in the study. This type of sampling allows the selection of individuals that provide relevant information to the research question [41]. The adopted sampling strategy ensured the inclusion of PTs from the various participating ACES, with an expected variety of viewpoints and heterogeneity in terms of professional experience, allowing the identification of barriers and facilitators from the different healthcare contexts. All PTs from the different ACES where implementation of the intervention will take place participated in the focus groups.

PTs were sent a written invitation letter and a study information sheet containing details about the study. All invited participants accepted to participate in the study and were sent a link giving them access to an informed consent form and instructions so they could keep a copy for themselves.

### Data collection

All participants were asked to fill out a sociodemographic questionnaire to collect the sample characteristics, including gender, age, academic qualifications, years of experience, years working in primary care and place of practice.

A semi-structured interview schedule was developed, exploring potential barriers and facilitators to PTs’ implementation of a behaviour change-informed exercise intervention. The interview schedule’s questions were informed by the COM-B model and the TDF (Additional file 2), using existing guidance [33, 37]. The semi-structured interview schedule was tested in a focus group with master’s students and minor modifications were made.

Two focus groups with PTs were performed. To ensure the quality and validity of the qualitative results, each focus group was composed of 7 participants [42]. Both focus groups were moderated by two female researchers. The moderator (CC, PhD) has a wide experience in conducting focus groups interviews and the co-moderator (STD, PhD candidate) received previous training and was responsible for taking notes throughout the discussions. Some participants already knew the moderator due to previous participation in other research projects. The project’s principal investigator (EBC) was also present during the focus groups but kept his camera turned off and did not speak throughout the whole discussions. The focus groups were held through videoconference, lasted approximately 90 min, and were audio and video recorded. The structure of the focus groups followed the recommendations by Finch et al. (2014) [42]: 1) scene setting and ground rules; 2) individual introduction; 3) opening topic; 4) discussion; 5) ending discussion.

At the start of each focus group, the moderator explained to participants the aims of the study and the research team’s interest in conducting the study, why they were selected and what would happen to the collected data. Then, the goals and the main evidence-based components of an intervention aimed at preventing recurrences of LBP were briefly introduced. The presentation included the following topics: 1) The importance of adopting regular exercise practice for the prevention of recurrences of LBP (for this study, regular exercise practice was considered as exercise that is performed on a regular basis, with exercise being defined as “a subcategory of physical activity that is planned, structured, repetitive, and purposeful in the sense that the improvement or maintenance of one or more components of physical fitness is the objective” [43]; 2) the need to structure (duration, number of sessions, and mode of application) and tailor the exercise plan to the patient's individual physical fitness needs (i.e., aerobic capacity, trunk and lower limb muscle resistance, motor control and flexibility); 3) the need to use behaviour change techniques (BCTs) to facilitate the adoption of regular exercise practice; and 4) the use of motivational interviewing principles to guide the whole intervention. Additionally, participants were presented a preliminary structure of the behaviour change-informed exercise intervention. This intervention would be composed of 24 exercise sessions, over the course of 12 weeks, with two 60-min sessions per week. One session would be supervised face-to-face by a physiotherapist and focused on the discussion of specific barriers and facilitators to exercise practice and on performing the exercise plan, while the other session would be performed by the patient autonomously as a home-based exercise session. The face-to-face session could be performed individually or in a group setting, where each patient would perform their own tailored exercise plan.

### Data management and analysis

Prior to the analysis of the focus groups, recordings were transcribed verbatim and anonymised with a pseudonym for each participant, by two researchers (AM and STD). A third researcher (CC) then checked the transcripts for accuracy.

A deductive content analysis was independently performed by two researchers (AM and STD), while a third researcher (CC) was approached to settle disagreements. The deductive content analysis was performed using a coding matrix based on the TDF domains and the COM-B components (Additional file 3) and was guided by the study’s aims of identifying barriers and facilitators to PTs’ implementation of a behaviour change-informed exercise intervention to promote the adoption of regular exercise practice. Microsoft Excel® was used to aid the organisation and analysis of the qualitative data.

Quotes selected to be used in the main manuscript were translated from European Portuguese to the English language by one researcher (AM). Following this, three other researchers (STD, CC and EBC) checked if the translation was accurate, culturally appropriate and conceptually equivalent to the source material. All researchers were fluent in the English language.

### Data trustworthiness

To ensure the quality and trustworthiness of the qualitative data, the credibility, transferability, dependability and confirmability criteria were considered [44].

Regarding the credibility criteria, the strategies of triangulation and member checking were used [44, 45]. All data was analysed independently by more than one researcher, who then compared results and discussed existing divergences, until consensus (investigator triangulation). Emerging barriers and facilitators were also shared with participants for feedback, allowing them to analyse the findings, as well as provide further information or clarification if they so intended (member checking).

Transferability requires the provision of a “thick description” of the setting in which the study was carried out, as well as of the participants. This allows outsiders to judge if the study findings are transferable to their own settings and contexts [44]. In this study, transferability was ensured by describing the context and setting in which the research was conducted, recruitment and participants’ characteristics.

The audit trail strategy was used to guarantee the dependability and confirmability criteria [44]. Dependability refers to the aspect of consistency and means that the research follows the accepted standards for a particular study design, while confirmability relates to neutrality and ensures that the findings are based on the data and not on the researchers’ subjective interpretations [44]. The audit trail was ensured through the description and documentation of all data collection and data analysis processes.

## Results

### Sample demographics

In total, 14 PTs participated in the focus groups. Participants had a mean age of 44.36 years old (± 10.75), were mostly of the female gender (*n* = 12, 86%), 10 (71%) had a graduate degree and four (29%) had a master’s degree. Sociodemographic characteristics are described in Table 1, while the characteristics of each individual participant are reported in Additional file 4.

**Table 1:** Sociodemographic characteristics of participating PTs

	**Total**
**Gender [n (%)]**	
Female	12 (86%)
Male	2 (14%)
**Age**	
Mean ± Standard deviation (SD)	44.36 years old ± 10.75
Minimum	30 years
Maximum	59 years
**Academic Qualification**	
Graduate degree	10 (71%)
Master’s degree	4 (29%)
**Years of Experience**	
Mean ± SD	21.79 years ± 10.58
Minimum	8 years
Maximum	38 years
**Years working in primary healthcare**	
Mean ± SD	8 years ± 11.71
Minimum	1 year
Maximum	26 years
**ACES [n (%)]**	
ACES Alentejo Central	3 (21%)
ACES Almada-Seixal	3 (21%)
ACES Arco Ribeirinho	5 (37%)
ACES Arrábida	3 (21%)

### Barriers to PTs’ implementation of a behaviour change-informed exercise intervention to promote the adoption of regular exercise practice

The deductive content analysis revealed 13 barriers to PTs’ implementation of a behaviour change-informed exercise intervention to promote the adoption of regular exercise practice in patients at risk of recurrence of LBP. These barriers were linked to four COM-B components (i.e., *Psychological Capability*; *Social Opportunity*; *Physical Opportunity*; and *Reflective Motivation*) and seven TDF domains (i.e., *Knowledge*; *Skills*; *Social influences*; *Environmental context and resources*; *Social/professional role and identity*; *Beliefs about capabilities*; and *Optimism*). Findings about the barriers and their classification within the COM-B components and TDF domains are summarised below, with some illustrative quotes throughout the text. Additional quotes for each barrier are provided in Table 2.

**Table 2:** Synthesis of PTs’ barriers identified from the focus groups

COM-B components	TDF domains	Barriers	Participants	Excerpts
Psychological Capability	Knowledge	Lack of knowledge on the risk factors for and management of recurrences of LBP, and BCTs	*PT5; PT6; PT7*	*“From my experience, I think one of the most common risk factors is the family socioeconomic status (…) Now, recommendations for the prevention of new episodes, I find it more difficult to… I think I would need to think a little bit more about that” (PT6, FG1)*
	Skills	Lack of skills for the implementation of the intervention	*PT2; PT4; PT10; PT11; PT13*	*“The tailored exercise prescription, I don’t feel comfortable (…) Exercise prescription, I honestly think I still don’t have tools strong enough to be able to… to be able to apply it.” (PT4, FG1)*
Social Opportunity	Social influences	Lack of peer interaction and discussions	*PT10*	*“(…) we work a lot alone. We end up not being able to share in loco with anyone, nor to clear a doubt that arises at that moment, the best strategy (…)” (PT10, FG2)*
Physical Opportunity	Environmental context and resources	Lack of time to implement and schedule incompatibilities between patients’ and primary healthcare’s schedules	*PT2; PT4; PT9; PT13*	*“We only work 7 hours, right? (…) it will be my pleasure to participate, but I’m just thinking about the daily time that we have, we don’t stop, right?” (PT4, FG1)*
		Existence of other priorities from their contexts (COVID-19 pandemic and vaccination)	*PT3; PT13*	*“(…) for two years we have been trying to get the ACES to carry out the implementation of other interventions and it still hasn’t been implemented. (…) things haven’t been easy and now with the vaccination problem, first it was the pandemic, now it’s the vaccination.” (PT3, FG1)*
		Lack of organisational support, that does not consider the intervention a priority	*PT3; PT7*	*“In our case, it has to do with the organisation of the ACES, right? That doesn’t even allow us to implemente it (…)” (PT7, FG1)*
		Lack of human and material resources	*PT6; PT7; PT8*	*“(…) I understand what all the colleagues have been saying, because we really are very few physiotherapists in the health centers.” (PT6, FG1)*
		Current focus on treatment rather than prevention	*PT9*	*“To my workplace, the priority is maybe very low (…) what I mean is interventions focused on prevention are not a priority (…) the priority is to solve the problem of that person who is sick and needs a physiotherapy intervention at that moment.” (PT9, FG2)*
		Low number/lack of patient referrals	*PT5; PT8; PT9; PT12*	*“So, in my case, I work in a health center a little bit more… geographically a little further away from the center and in my case, I almost have no referrals (…) It’s one of my handicaps.” (PT5, FG1)*
		Unable to implement other interventions	*PT1; PT3; PT7; PT13*	*“Then, about my workplace, you must have already noticed, right? If I’m not even able to implement other interventions…” (PT13, FG2)*
Reflective Motivation	Social/professional role and identity	Practice according to a paternalistic model of care that does not promote patient autonomy	*PT13*	*“(…) but in general, we still have a lot of control over the patients (…) We often say to them, or we tell each other, that they don’t do anything for themselves, but maybe many of us don’t do anything so that the patients understand this.” (PT13, FG2)*
	Beliefs about capabilities	Lack of confidence for the implementation of the intervention	*PT1; PT2; PT4; PT5; PT7; PT8; PT11*	*“Then there’s also another issue that worries me, which is the difficulty in changing behaviors (…) and I've been having that difficulty (…) " (PT8, FG2)*
	Optimism	Pessimism about the implementation of the intervention	*PT7*	*“(…) so, I really want to implement. My wish is to implement, without a doubt, but since other interventions still haven’t been implemented… I’m not very, I don’t have a lot of expectations (…)” (PT7, FG1)*

Within the COM-B component *Psychological Capability*, two barriers related to the TDF domains of *Knowledge* and *Skills* were identified. Participants demonstrated a general lack of knowledge on the potential risk factors and management recommendations for recurrences of LBP and BCTs. They were also unanimous about their lack of skills to implement the behaviour change-informed exercise intervention, and the need for training in its several components.

*“(…) what I feel is most lacking is, without a doubt, the issues of behaviour change (…) being something that I never applied in my clinical practice, it’s without a doubt, what I need more help with.” (PT11, FG2)*

*“Techniques? [BCTs] No, well, I don’t know what you mean by techniques.” (PT7, FG1)*

Within the scope of the COM-B component *Social Opportunity*, the analysis revealed one barrier at the level of the TDF domain *Social influences*. This barrier was related to PTs’ inability to discuss and interact with their peers, given the fact that many of them work alone, and are therefore isolated from other colleagues. The most commonly identified COM-B component and TDF domain were *Physical Opportunity* and *Environmental context and resources*, respectively. In total, seven barriers were identified. Participants identified incompatibilities between patients’ and primary healthcare schedules, as well as a lack of time in their contexts which could hinder their ability to effectively implement the intervention. This lack of time was mainly justified by the existence of other competing activities, little time to treat patients and the high number of referrals to other existing interventions.

*“Because, in one healthcare unit, I have a high number of referrals (…) and the availability in terms of time… it’s a bit short to start another intervention, still having other patients on waitlist. It’s just more in that sense of time.” (PT9, FG2)*

The existence of other priorities from their contexts, specifically the panorama created by the COVID-19 pandemic was also highlighted as a barrier. Additionally, some participants felt that the coordinators of their primary healthcare centres did not consider physiotherapy issues to be a primary concern and did not consider this type of interventions a priority. Participants stated that their healthcare centres placed increased focus of human resources on the treatment of acute conditions, rather than prevention. Other context-related barriers were reported, such as the low number or even lack of patient referrals and the inability to implement other interventions.

*“The only issue is that we have been trying for two years for the ACES to carry out the implementation of another intervention, and there is no way (…) things have not been simple (…)” (PT3, FG1)*

The low number of PTs currently working in primary healthcare and the lack of material resources in some primary healthcare centres were also outlined as possible barriers to the implementation of the intervention.

*“And even material resources (…) There are practically none. For example, to work in a group, in one unit where I am, there isn’t… there’s only one ball. So, no… you can’t make certain group activities.” (PT8, FG2)*

In the COM-B component of *Reflective Motivation*, three barriers were identified. Power differences between PTs and patients were outlined, as it was considered that PTs practice mainly according to a paternalistic model, that does not promote patient autonomy. This barrier was linked to the TDF domain of *Social/professional role and identity*. A barrier related to the TDF domain *Optimism* was identified. Pessimism regarding the implementation was demonstrated, justified by low expectations that the implementation of the intervention will happen, given the fact that other interventions still have not been implemented. Finally, one barrier within the scope of the TDF domain *Beliefs about capabilities* was identified. Some participants reported lack of confidence for the implementation of a behaviour change-informed exercise intervention, associated with doubts about their capabilities to implement and their ability to respond to the rising needs, not having been able to previously implement other interventions in their healthcare centres, and not having the necessary skills to effectively implement the intervention.

*“My confidence level for implementation is low at the moment (…) in terms of confidence, I don’t feel very confident, and I believe that training would help a lot, of course.” (PT1, FG1)*

### Facilitators to PTs’ implementation of a behaviour change-informed exercise intervention to promote the adoption of regular exercise practice

Regarding the facilitators to PTs’ implementation of a behaviour change-informed exercise intervention to promote the adoption of regular exercise practice in patients at risk of recurrence of LBP, 23 were identified. These were linked to five COM-B components (i.e., *Psychological Capability*; *Social Opportunity*; *Physical Opportunity*; *Reflective Motivation*; and *Automatic Motivation*) and 13 TDF domains (i.e., *Knowledge*; *Skills; Memory, attention and decision processes*; *Behavioural regulation*; *Social influences*; *Environmental context and resources*; S*ocial/professional role and identity*; *Beliefs about capabilities*; *Beliefs about consequences*; *Optimism*; *Intentions*; *Reinforcement*; and *Emotion*). Findings related to the facilitators and their classification within the COM-B components and TDF domains are summarised below. Some representative quotes are provided within the text, while additional quotes for each facilitator are presented in Table 3.

**Table 3:** Synthesis of PTs’ facilitators identified from the focus groups

COM-B components	TDF domains	Facilitators	Participants	Excerpts
Psychological Capability	Knowledge	Having knowledge on the recommendations for the management of recurrences of LBP	*PT5*	*“How to manage the pain, if they should move, which movements to perform… when they should seek a health professional, if they should seek them, well, it’s discussing these issues with the patient.” (PT5, FG1)*
	Skills	Acquisition of skills through participation in the implementation of the intervention	*PT3; PT4*	*“(…) well, it will allow us to have more theoretical skills, in the sense of changing… as I said, I was just talking about this, about behaviour change. Lead patients to that change (…)” (PT8, FG2)*
	Memory, attention and decision processes	The interventions is aligned with current practice	*PT9; PT10*	*“(…) I think that for those who have been in primary healthcare for a few years, promoting physical activity habits, this is already a little bit of what we do.” (PT10, FG2)*
		Positive past experiences related to participation in exercise interventions	*PT3; PT4; PT6*	*“I think that the knowledge that we’ve previously acquired through the implementation of other exercise interventions can also help us now with this new one (…)” (PT6, FG1)*
	Behavioural regulation	Ability to organise and manage work activities according to the needs and availabilities	*PT1; PT6; PT7*	*“(…) if I know exactly who has priority to enter in, in this new intervention, right? For example, which patients do I have to prioritise? I may have to prioritse this one, next week I’ll prioritise two other people.” (PT6, FG1)*
Social Opportunity	Social influences	Therapeutic relationships previously established	*PT6*	*“(…) I think there will be a lot of patient adherence (…) They already trust us, and many patients already know us… no, I think that adherence will be easier.” (PT6, FG1)*
		Professional relationships and collaboration between physiotherapists	*PT6*	*“(…) as long as we’re not… isolated, each one doing different things (…) I think we need this structure and being more together and having more strength together (…)” (PT6, FG1)*
		Relationship with and involvement of the multidisciplinary team	*PT4; PT6; PT8; PT10; PT11*	*“(…) I know that an intervention of this nature will create even more trust from the other professionals in us, trust that we are capable…” (PT6, FG1)* *“Since the patients also see their doctors more often, maybe, having a partnership with the doctor (…) to again transmit this message that’s important to continue performing exercise (…).” (PT8, FG2)*
Physical Opportunity	Environmental context and resources	Context provides the necessary time to implement the intervention	*PT1; PT6; PT12*	*“This on the assumption that I would have 2 hours a week dedicated to this intervention, right? Or I can have more, right? It depends on our ability to manage or if our management is flexible, which it is in my case (…)” (PT6, FG1)*
		Having organisational support, that consider the intervention a priority	*PT10*	*“I’d just like to say that here in my ACES this intervention has a high priority at the organisational level (…)” (PT10, FG2)*
		The intervention’s principles are aligned with primary healthcare’s principles	*PT14*	*“(…) we already are in a context that is based on this principle of delegating competences to the patients over their own health.” (PT14, FG2)*
		High number of referrals	*PT8*	*“I’m in a unit where I have lots of referrals and I even have a wait list for patients for other interventions.” (PT8, FG2)*
		Need for few resources	*PT12*	*“… in terms of resources (…) it’s not something that involves having a lot of things. So, having us, the person, and a place to do the exercises is enough.” (PT12, FG2)*
		High incidence of recurrences of LBP, which justified the need for the intervention^a^	*PT2*	*“(…) here in our context it’s a common condition… low back pain is very recurrent, we have many referrals… especially many referrals which are a first episode, which are already recurring episodes.” (PT2, FG1)*
Reflective Motivation	Social/professional role and identity	Benefits for the physiotherapists and for the profession	*PT1; PT2; PT3; PT4; PT5; PT6; PT9; PT10; PT12*	*“Our work is very under-recognised in primary care, and we must grab on to something that makes us different from the others and united to each other. I think that’s very important. (…)” (PT3, FG1)*
		The intervention aligns with physiotherapists’ professional identity and role in primary healthcare	*PT1; PT5; PT9; PT10*	*“This type of interventions (…) we have a role of changing behaviours, which is something that I also identify with and me working in primary healthcare is exactly for this… to change behaviours (…)” (PT9, FG2)*
	Beliefs about capabilities	High confidence levels for the implementation of the intervention	*PT6; PT10; PT11; PT14*	*“(…) on the assumption that we will have training, right? So, my confidence level is based on that assumption. I think my confidence level is very high.” (PT6, FG1)*
	Optimism	Optimism about the implementation of the intervention	*PT6; PT11; PT13*	*“(…) I believe that after the person starts seeing the benefits, it will certainly be easier for them to continue” (PT11, FG2)*
	Beliefs about consequences	Beliefs about the potential patient benefits and improvement of quality of care	*PT2; PT6; PT8; PT9; PT10; PT11; PT12; PT13; PT14*	*“I think that the main benefit would be… them gaining exercise habits. It’s that this intervention will help them gain exercise habits on a regular basis.” (PT10, FG2)* *“(…) also, in terms of medication, if they know the strategies that they can do to… to prevent recurrences, they can also reduce medication. It’s another benefit, for example.” (PT8, FG2)*
	Intentions	Willingness to change clinical practice	*PT1; PT3; PT4; PT5; PT6; PT7; PT9; PT10; PT13*	*“(…) I think that I really consider it to be a top priority for me (…) to find a way in my schedule to fit this type of intervention (…)” (PT6, FG1)*
Automatic Motivation	Reinforcement	Joint development of the interventions with higher education institutions	*PT5; PT13*	*“First because we have these education institutions supporting the program (…) Credible, in several places at the same time, with well-studied, well-structured data… I think this isn’t very common in physiotherapy and we really needed it.” (PT13, FG2)*
		Continuity of care	*PT8; PT11*	*“I think that it’s very relevant to implement this intervention, because it’s also the reinforcement of what has been done before (…)” (PT8, FG2)*
	Emotion	Positive emotions about the implementation of the intervention	*PT13*	*“Because it’s an area that I like (…) I’ve worked a lot with patients with low back pain before, not now, but… I would like to do it again” (PT13, FG2)*

^a^Non-modifiable facilitator

In the COM-B component *Psychological Capability*, five facilitators were identified within the scope of the TDF domains of *Knowledge*, *Skills*, *Memory, attention and decision processes*, and *Behavioural regulation*. Conversely to the lack of knowledge previously reported, having some degree of knowledge about the recommendations for the management of recurrences of LBP was identified as a facilitator. The implementation of the intervention was also associated with participation in a future training programme, which was expected to promote the acquisition and development of skills that would allow PTs to improve the care provided and add value to their practices.

*“(…) this intervention will fill those big gaps that we have in primary care, and give us new knowledge, right? (…) technical and scientific knowledge to be applied in our practices and actually have this reliable demonstration of our intervention.” (PT4, FG1)*

PTs reported overall positive previous experiences with exercise interventions that took place within their primary healthcare centres, and which included exercise and even behaviour change components. These experiences were considered to be an advantage for the implementation of the behaviour change-informed intervention. Another important aspect was PTs’ ability to organise and manage their work activities according to the rising needs and availabilities, allowing them to introduce a new intervention into their schedules. Additionally, some participants perceived that this kind of interventions were, in a way, similar to their current practice. They mentioned having already implemented interventions focused on the promotion of physical activity behaviours, but not in a structured and measurable way.

*“(…) I confess that I already end up doing a little bit of this, but not in a measurable way. So, that’s what maybe we’ll start doing. It’s doing something that is measured and that we can apply.” (PT9, FG2)*

Regarding the COM-B component of *Social Opportunity* and the TDF domain of *Social influences*, the analysis revealed three facilitators. Therapeutic relationships previously established with patients in other interventions were seen as an enabling factor for patient adherence. Furthermore, participants considered positive relationships and interdisciplinary work between healthcare professionals, as well as their involvement with this type of interventions as essential aspects, potentially having a positive effect on the way physiotherapy is regarded and perceived. Just as the relationships established with patients and other healthcare professionals, professional relationships and collaboration between PTs were also outlined as key factors. The inclusion of and cooperation between PTs from different primary healthcare centres in the development of the intervention was deemed to contribute to possibly having a more structured practice and a stronger profession.

*“(…) we could involve the remaining health team because patients also come to nursing consultations… they have nutrition, psychology consultations. And I think that if we can explain this cause to the colleagues and ask them to help us remind the patient of the importance of maintaining these exercises (…) (PT10, FG2)*

For the COM-B component *Physical Opportunity* and the TDF domain *Environmental context and resources*, six facilitators were identified. It was considered relevant to develop and implement this kind of intervention to promote the adoption of regular exercise practice in patients at risk of recurrence of LBP, as it would target a health condition with high impact and would complement the service provided by other interventions for the management of patients with LBP episodes. Having support from the healthcare centres’ coordination, who considered this type of interventions a priority, was also considered a critical factor for the implementation of the intervention. Other identified facilitators were the perception that this type of intervention is aligned with primary healthcare’s principles and having a high number of referrals of patients with LBP, which constitute possible future participants for the new intervention. Additionally, even though the lack of material resources was identified as a barrier, it was also pointed out that the implementation of this kind of interventions would not require a high number of resources, thus the need for few resources was considered a facilitator to the implementation. Some participants reported that their contexts provided the necessary time to implement a new intervention. Their contexts allowed them to freely manage their schedules, but they were conscient that this may not be a reality for many of the other colleagues.

*“... at least… we manage our schedules (…) so, in terms of time, it depends on the number of referrals and then if we have other requests, but it’s not a constraint, nor is something too difficult.” (PT12, FG2)*

Within the COM-B component of *Reflective Motivation*, six facilitators were also identified. Two facilitators were linked to the TDF domain *Social/professional role and identity*. Participants indicated possible emerging benefits from the implementation of a behaviour change-informed exercise intervention, such as expectations of professional recognition, differentiation and a chance for professional development. They also considered the intervention’s principles to be aligned with their professional identity and with their role as healthcare professionals in primary healthcare.

*“If I think that the intervention is very interesting and seems to me to meet the principles of what our role as physiotherapists is in primary health care? Of course, I have no doubts about that.” (PT5, FG1)*

A facilitator related to perceived high levels of confidence for the implementation was linked to the TDF domain *Beliefs about capabilities*. While some participants demonstrated a perceived lack of confidence to implement the intervention (reported in the barriers), others reported high confidence levels. These confidence levels were dependent on their motivation levels to implement, the acquisition of knowledge and skills through participation in a training programme, and the ability to promote patient adherence to the intervention.

*“So, supposedly, the patients will already be predisposed to participate and already know the intervention, right? (…) They are already willing to adhere to an intervention that was already presented to them. I think that our role is to help them continue, I think so, I would score a very high level of confidence for the implementation.” (PT10, FG2)*

One facilitator was found within the TDF domain of *Optimism*. In contrast with what was reported in the barriers, some participants were optimistic about the potential implementation of a new behaviour change-informed exercise intervention, the possible improvements to their practices, the possible benefits at the patient level and hopes of a “paradigm shift” in physiotherapy practice.

*“Each patient treats himself, either by going to treatment or with the things they do at home, and... I think these interventions also help us... we keep saying this so many times to the patient, it may be that it also gets into our own heads.” (PT13, FG2)*

Participants considered the intervention a priority and expressed willingness to change their practice, and this facilitator was classified in the TDF domain of *Intentions*. Most PTs also anticipated and demonstrated beliefs about the potential benefits of the intervention for the patients and the improvement of quality of care, and this was linked to the TDF domain *Beliefs about consequences*. Participants consensually identified patient benefits in the development of self-regulation capability, increased patient confidence and autonomy to manage their musculoskeletal health, prevent recurrences of LBP, and reduce unnecessary healthcare consumption. Simultaneously, the acquisition of skills to manage a possible recurrence was also identified as an important aspect. Additionally, gaining exercise practice habits, reducing medication intake, increasing work productivity and improving quality of life were also pointed out by PTs as potential patient benefits of this kind of interventions.

*“(…) a great benefit of this intervention may be related to patients themselves gaining more confidence in their skills and abilities, right? (…) because that's the only way they may not come back here to… resort so much to healthcare services, right? (…)” (PT6, FG1)*

*“If they have less low back pain, they will be able to spend more time at work, have fewer sick leaves, they will have a better quality of life (…)” (PT11, FG2)*

Lastly, three facilitators were found for the COM-B component *Automatic Motivation*. One facilitator was tied with the TDF domain *Emotion* and was related to the demonstration of positive emotions about the future implementation of the intervention. The other two facilitators were linked with the *Reinforcement* domain. The development of these interventions through a partnership with a research team, tied to several higher education institutions, was also considered to be an important aspect, giving validity and legitimacy to the intervention and physiotherapy practice, and increasing the visibility of the results achieved.

*“(...) I think that the fact that we are articulated with accredited higher education institutions, that are doing a research project… it also legitimises our work, giving us more visibility (…)” (PT5, FG1)*

The perception that this type of interventions will allow for a continuity of care, reinforcing what had been previously done in other interventions for the management of LBP patients and providing the opportunity to continue following patients after they are discharged was also highlighted as an important enabler.

*“(…) I think that the intervention is very relevant and important, and we’re missing something like this, right? We, who treat people with low back pain, feel like we’re missing something more to offer people after they’re discharged.” (PT11, FG2)*

## Discussion

The purpose of this study was to explore PTs’ perceived barriers and facilitators to the implementation of a behaviour change-informed exercise intervention to promote the adoption of regular exercise practice in patients at risk of recurrence of LBP.

The COM-B model and the TDF provided a detailed understanding of the barriers and facilitators that may impact the future implementation of a behaviour change-informed exercise intervention by PTs. The analysis revealed a total of 13 barriers and 23 facilitators to PTs’ target behaviour, classified within five COM-B components and 13 TDF domains. Some of the results of this study are in line with previously identified barriers and facilitators in the literature, related to implementation science or implementation of other interventions in primary healthcare.

This study found that PTs lack knowledge and skills for the implementation of a behaviour change-informed intervention, specifically the integration of BCTs in their practice. However, they recognised their importance in promoting patients’ behaviour change and identified the acquisition of knowledge and skills through training and participation in the implementation of the intervention as facilitators. These findings are corroborated by a previous systematic review that indicates that PTs do not feel adequately trained to use psychologically informed interventions in their clinical practices [46]. Furthermore, gains such as enhancement of skills, knowledge and participation in research projects have been identified as implementation facilitators [47].

Several context-related barriers and facilitators were identified. The existence of other priorities from the contexts and not having organisational support were considered by participants as one of the most important barriers to the future implementation of a behaviour change-informed exercise intervention. Conversely, some participants identified having the necessary organisational support from coordination, who considered the intervention and its implementation a priority. These divergent perspectives may be explained by PTs’ previous experiences in implementing or trying to implement other interventions, where some had organisational support, while others did not. These results are in line with what has been previously identified in a systematic review that aimed to identify the causes of the evidence-to-practice gap for complex interventions in primary healthcare, in which having or not having support was identified either as a barrier or facilitator, respectively [48]. Other determinants, namely lack or presence of time and resources or alignment between the intervention’s and primary healthcare principles have also been identified in other studies [47–51].

The findings of this study show that PTs lack opportunities to interact and discuss with their peers, while the establishment of relationships with patients, other PTs or members of the multidisciplinary team were considered to be potential enablers to the delivery of the intervention. This has also been previously reported in the literature, in which interdisciplinary work and the presence of a positive and trusting inter-professional relationship, the opportunity to discuss issues and challenges, and relationships with other healthcare professionals and patients have also been identified to positively influence implementation [48, 50].

It was identified that most PTs still practice according to a paternalistic model of care that does not promote patient autonomy. This is in line with previous findings that suggest that Portuguese PTs seem to favour a reasoning and practice approach more consistent with a traditional biomedical model of care and a practice that is mostly clinician-centred [52]. Potential benefits, such as the development of the profession and the alignment of the intervention with PTs’ professional identity and role in primary healthcare have also been found to be an important factor for implementation [47].

Levels of self-efficacy for the implementation of the intervention were also an important aspect raised by PTs, with some being confident, while others were not. This confidence was mediated by several factors, from previous experiences with implementation of other interventions to PTs’ own levels of motivation and perception regarding their ability to effectively deliver the intervention, among others. This factor has also been identified to either hamper or facilitate the implementation of interventions [47, 48], and might be an important target since there is evidence of PTs improving their confidence after participation in training programmes [53, 54].

Facilitators, such as positive feelings and emotions related to the capability to implement and to patients’ experienced benefits and motivation to improve the care provided have been described by other studies [47, 50]. In the present study, these aspects were also identified, with participants demonstrating positive emotions towards the potential implementation of the intervention and beliefs about the possible patient benefits and the improvement of the quality of care provided.

Based on the results of this study, it will be possible to develop a training programme to support PTs’ implementation of the behaviour change-informed exercise intervention. This training programme will target the specific determinants identified by PTs, aiming to promote not only their capability (e.g., knowledge on recurrences of LBP and development of skills on exercise prescription and use of BCTs) for implementation, but also promote aspects related to their motivation (e.g., increase their confidence for delivering the intervention) and opportunity (e.g., promote interaction with other peers and brainstorming possible ways to overcome contextual barriers hampering implementation).

### Strengths and limitations

The use of the BCW, including the COM-B model and the TDF, first allowed to identify specific barriers and facilitators and focus on what needs to happen for PTs’ implementation of a behaviour change-informed exercise intervention. Through the COM-B model of behaviour, it was possible to first conceptualise the findings of this study within participants’ capability, opportunity and motivation, while the TDF then allowed for a more comprehensive and specific understanding of the different barriers and facilitators. The information gathered in this study, which corresponds to the BCW step of identifying what needs to change, will be used to inform the development of an intervention aimed at PTs. The modifiable determinants will be selected and as outlined by the BCW, the next steps will be to identify the intervention options (i.e., intervention functions), content (i.e., behaviour change techniques) and implementation options (i.e., modes of delivery). This whole process will be described in a subsequent study.

Some limitations of the study need to be considered. Social desirability bias, the desire to conform to social acceptability, may have been present and may have influenced participants’ responses during the focus groups. This bias might have been further strengthened by the fact that some participants knew the moderator and by the presence of the principal investigator during the focus groups. Furthermore, all possible barriers and facilitators to the implementation of the behaviour change-informed exercise intervention may not have been identified, since the perspectives of other important stakeholders, such as ACES coordinators were not explored.

## Conclusions

The findings of this study highlight a wide range of barriers and facilitators to PTs’ implementation of a behaviour change-informed exercise intervention to promote the adoption of regular exercise practice in patients at risk of recurrence of LBP. Using the BCW, including the COM-B model and the TDF, it was possible to identify a total of 13 barriers (4 COM-B components and 7 TDF domains) and 23 facilitators (5 COM-B components and 13 TDF domains). Some barriers, such as lack of skills and confidence to implement were expected as it is a novel intervention for all participants. Other determinants seemed to be identified by PTs with different practice profiles. Those with previous experience in the implementation of similar health interventions tended to mention a wide range of facilitators, while those who failed to implement comparable interventions focused on identifying organisational barriers. Based on these findings, and using the remaining stages of the BCW, it will be possible to develop a behaviour change-informed intervention for PTs, aimed at targeting the identified barriers and facilitators and supporting them in the successful implementation of the behaviour change-informed exercise intervention.

### Supplementary Information

**Additional file 1. **COREQ (COnsolidated criteria for REporting Qualitative research) checklist.

**Additional file 2. **Semi-structured interview guide developed for the focus groups.

**Additional file 3. **Coding matrix developed for the analysis of the focus groups transcripts.

**Additional file 4.** Sociodemographic characteristics of each individual participant.

## Data Availability

The dataset that was generated and/or analysed during the current study is not publicly available due to ethical reasons. Data are however available from the corresponding author upon reasonable request.

## Abbreviations

ACES: Agrupamentos de Centros de Saúde (Groups of primary healthcare centres)
BCW: Behaviour Change Wheel
BCTs: Behaviour Change Techniques
COM-B: Capability, Opportunity, Motivation - Behaviour
COREQ: The Consolidated Criteria for Reporting Qualitative Research
FG: Focus group
LBP: Low Back Pain
PTs: Physiotherapists
RHAs: Regional Health Administration
RHAA: Regional Health Administration of Alentejo
RHALTV: Regional Health Administration of Lisbon and Tagus Valley
TDF: Theoretical Domains Framework
YLDs: Years Lived with Disability
