# Correction: Physiotherapists’ barriers and facilitators to the implementation of a behaviour change-informed exercise intervention to promote the adoption of regular exercise practice in patients at risk of recurrence of low back pain: a qualitative study

**DOI:** 10.1186/s12875-024-02333-4

**Published:** 2024-03-22

**Authors:** Alexandre Moniz, Susana T. Duarte, Pedro Aguiar, Carmen Caeiro, Diogo Pires, Rita Fernandes, Diogo Moço, Marta M. Marques, Rute Sousa, Helena Canhão, Jaime Branco, Ana Maria Rodrigues, Eduardo B. Cruz

**Affiliations:** 1 Comprehensive Health Research Center (CHRC), NOVA Medical School|Faculdade de Ciências Médicas, NMS|FCM, Universidade NOVA de Lisboa, Lisbon, Portugal; 2 EpiDoc Unit, NOVA Medical School|Faculdade de Ciências Médicas, NMS|FCM, Universidade Nova de Lisboa, Lisbon, Portugal; 3 Departamento de Fisioterapia, Escola Superior de Saúde, Instituto Politécnico de Setúbal, Setúbal, Portugal; 4 Comprehensive Health Research Center (CHRC), National School of Public Health, Universidade NOVA de Lisboa, Lisbon, Portugal; 5 National School of Public Health, Universidade NOVA de Lisboa, Lisbon, Portugal; 6 Comprehensive Health Research Center (CHRC), Universidade NOVA de Lisboa, Lisbon, Portugal; 7 Serviço de Reumatologia Do Hospital Egas Moniz, Centro Hospitalar Lisboa Ocidental (CHLO), Lisbon, Portugal; 8 Rheumatology Unit, Hospital Dos Lusíadas, Lisbon, Portugal

**Correction: BMC Prim Care** **25, 39 (2024)**

10.1186/s12875-024-02274-y

Following publication of the original article [1], the authors reported an error in the affiliations of author Marta M. Marques. The correct affiliations are provided below.

*Incorrect affiliations*:

Marta M. Marques^1,2,^

^1^Comprehensive Health Research Center (CHRC), NOVA Medical School|Faculdade de Ciências Médicas, NMS|FCM, Universidade NOVA de Lisboa, Lisbon, Portugal

^2^EpiDoc Unit, NOVA Medical School|Faculdade de Ciências Médicas, NMS|FCM, Universidade Nova de Lisboa, Lisbon, Portugal

*Correct affiliations*:

Marta M. Marques^4, 5^

^4^Comprehensive Health Research Center (CHRC), National School of Public Health, Universidade NOVA de Lisboa, Lisbon, Portugal

^5^National School of Public Health, Universidade NOVA de Lisboa, Lisbon, Portugal

The original article has been corrected.
