# Supplementary material for: Physiotherapists’ barriers and facilitators to the implementation of a behaviour change-informed exercise intervention to promote the adoption of regular exercise practice in patients at risk of recurrence of low back pain: a qualitative study
Source: BMC Prim Care. 2024 Jan 26;25:39. doi: 10.1186/s12875-024-02274-y (PMC10811813; doi:10.1186/s12875-024-02274-y)
Supplement: Supplementary file 2 — Additional file 2. Semi-structured interview guide developed for the focus groups. [file 12875_2024_2274_MOESM2_ESM.docx]

**Additional file 2.** Semi-structured interview guide developed for the focus groups

| **COM-B components** | **TDF domains** | **Questions** |
| --- | --- | --- |
| Psychological Capability **(PC)** | Knowledge **(K)** | Do you know why it is recommended that patients learn to prevent future episodes/manage new episodes of low back pain? |
|  |  | Do you know why the adoption of regular exercise practice it is recommended to prevent future episodes/manage new episodes of low back pain? |
|  |  | Do you know what to teach patients so that they know what to do in case of a new episode of low back pain? |
|  | Skills **(S)** | Do you feel that you have the necessary skills to implement an intervention to promote the adoption of regular exercise practice? |
|  |  | What skills do you think you need to develop in order to implement an intervention to promote the adoption of regular exercise practice? |
|  | Memory, attention and decision processes **(MADP)** | What are your previous experiences related to implementing and/or participating in interventions of this nature? |
|  | Behavioural regulation **(BR)** | Considering your current practice, what kind of existing practices can facilitate the implementation of the intervention? And make it difficult? |
|  |  | In your opinion, what could help you integrate the intervention into your routine practice? |
| Social Opportunity **(SO)** | Social influences **(SI)** | In what way does your work environment, including your colleagues, other professionals, coordination, promote and support the implementation of interventions of this nature? |
| Physical Opportunity **(PO)** | Environmental context and resources **(ECR)** | In what way does your work context favours/encourages the implementation of an intervention to promote the adoption of a regular exercise practice? |
|  |  | Do you consider that you have the necessary conditions (time and resources) to implement the intervention? |
| Reflective Motivation **(RM)** | Social/professional role and identity **(SPRI)** | In what way do you consider that the implementation of an intervention to promote the adoption of regular exercise practice is important for you, for your current practice, and for your profession? |
|  |  | To what extent is the implementation of this intervention something you feel you should be a part of? |
|  | Beliefs about capabilities **(BCap)** | How confident do you feel about implementing an intervention to promote the adoption of regular exercise practice? |
|  |  | How easy or difficult is it for you to participate in the implementation of the intervention? |
|  | Optimism **(O)** | In what way do you think that implementing an intervention to promote the adoption of regular exercise will be successful in your workplace? |
|  |  | In what way do you think this intervention could play an important role in preventing low back pain recurrences? |
|  | Beliefs about consequences **(BCon)** | What kind of benefits do you consider that an intervention to promote the adoption of regular exercise practice will bring to patients, both now and in the future? |
|  |  | In what way do you think that introducing the intervention into your routine practice will result in more benefits than costs? |
|  | Intentions **(I)** | Do you intend to implement an intervention to promote the adoption of regular exercise? If not, why not? Will you implement it with all patients that recovered from an episode of low back pain and at risk of recurrence? If not, why not? |
|  |  | What is the level of priority you give to the implementation of the intervention? Why? |
| Automatic Motivation **(AM)** | Reinforcement **(R)** | Do you feel that the implementation of an intervention to promote the adoption of regular exercise practice will bring you greater recognition in your work context? If no/yes, why? |
|  | Emotion **(E)** | When you implement an intervention based on the best available scientific evidence, how does that make you feel? Does this feeling make it easier or more difficult to perform the intervention? |
