# Supplementary material for: Physiotherapists’ barriers and facilitators to the implementation of a behaviour change-informed exercise intervention to promote the adoption of regular exercise practice in patients at risk of recurrence of low back pain: a qualitative study
Source: BMC Prim Care. 2024 Jan 26;25:39. doi: 10.1186/s12875-024-02274-y (PMC10811813; doi:10.1186/s12875-024-02274-y)
Supplement: Supplementary file 3 — Additional file 3. Coding matrix developed for the analysis of the focus groups transcripts. [file 12875_2024_2274_MOESM3_ESM.docx]

***Additional file 3.*** *Coding matrix developed for the analysis of the focus groups transcripts.*

| **COM-B components** | | **TDF domains** | **Barriers** | **Facilitators** |
| --- | --- | --- | --- | --- |
| 1. Capability | 1.1. Psychological | 1.1a. Knowledge | 1.1a– | 1.1a+ |
|  |  | 1.1b. Cognitive and interpersonal skills | 1.1b– | 1.1b+ |
|  |  | 1.1c. Memory, attention and decision processes | 1.1c– | 1.1c+ |
|  |  | 1.1d. Behavioural regulation | 1.1d– | 1.1d+ |
|  | 1.2. Physical | 1.2a. Skills | 1.2a– | 1.2a+ |
| 2. Opportunity | 2.1. Social | 2.1.a. Social influences | 2.1a– | 2.1a+ |
|  | 2.2. Physical | 2.2.a. Environmental context and resources | 2.2a– | 2.2a+ |
| 3. Motivation | 3.1. Reflective | 3.1a. Social/professional role and identity | 3.1a– | 3.1a+ |
|  |  | 3.1b. Beliefs about capabilities | 3.1b– | 3.1b+ |
|  |  | 3.1c. Optimism | 3.1c– | 3.1c+ |
|  |  | 3.1d. Beliefs about consequences | 3.1d– | 3.1d+ |
|  |  | 3.1e. Intentions | 3.1e– | 3.1e+ |
|  |  | 3.1f. Goals | 3.1f– | 3.1f+ |
|  | 3.2. Automatic | 3.2a. Reinforcement | 3.2a– | 3.2a+ |
|  |  | 3.2b. Emotion | 3.2b– | 3.2b+ |
