# Supplementary material for: Physiotherapists’ barriers and facilitators to the implementation of a behaviour change-informed exercise intervention to promote the adoption of regular exercise practice in patients at risk of recurrence of low back pain: a qualitative study
Source: BMC Prim Care. 2024 Jan 26;25:39. doi: 10.1186/s12875-024-02274-y (PMC10811813; doi:10.1186/s12875-024-02274-y)
Supplement: Supplementary file 4 — Additional file 4. Sociodemographic characteristics of each individual participant. [file 12875_2024_2274_MOESM4_ESM.docx]

**Additional file 4.** Sociodemographic characteristics of each individual participant

| **Patient** | **Gender** | **Age** | **Academic Qualification** | **Years of experience** | **Years working in primary healthcare** | **ACES** |
| --- | --- | --- | --- | --- | --- | --- |
| PT1 | Female | 31 | Graduate degree | 9 | 3 | ACES Alentejo Central |
| PT2 | Female | 31 | Graduate degree | 8 | 1 | ACES Alentejo Central |
| PT3 | Female | 59 | Graduate degree | 29 | 6 | ACES Almada Seixal |
| PT4 | Female | 53 | Master’s degree | 32 | 5 | ACES Arco Ribeirinho |
| PT5 | Female | 55 | Graduate degree | 34 | 3 | ACES Arco Ribeirinho |
| PT6 | Female | 52 | Master’s degree | 30 | 23 | ACES Arrábida |
| PT7 | Female | 50 | Graduate degree | 28 | 21 | ACES Almada Seixal |
| PT8 | Female | 40 | Graduate degree | 17 | 17 | ACES Arrábida |
| PT9 | Female | 41 | Master’s degree | 20 | 6 | ACES Alentejo Central |
| PT10 | Female | 49 | Graduate degree | 27 | 25 | ACES Arco Ribeirinho |
| PT11 | Male | 30 | Master’s degree | 8 | 4 | ACES Arrábida |
| PT12 | Female | 39 | Graduate degree | 15 | 14 | ACES Arco Ribeirinho |
| PT13 | Female | 59 | Graduate degree | 38 | 26 | ACES Almada Seixal |
| PT14 | Male | 32 | Graduate degree | 10 | 10 | ACES Arco Ribeirinho |
